# Beckmann Rearrangement of Ketoximes Induced by Phenyl Dichlorophosphate at Ambient Temperature

**DOI:** 10.3390/molecules171113662

**Published:** 2012-11-20

**Authors:** Chun-Wei Kuo, Min-Tsang Hsieh, Shijay Gao, Yi-Ming Shao, Ching-Fa Yao, Kak-Shan Shia

**Affiliations:** 1 Institute of Biotechnology and Pharmaceutical Research, National Health Research Institutes, Miaoli County 35053, Taiwan; 2 Department of Chemistry, National Taiwan Normal University, Taipei 116, Taiwan; 3 Chinese Medicinal Research and Development Center, China Medical University Hospital, 2 Yude Road, Taichung 40447, Taiwan

**Keywords:** Beckmann rearrangement, phenyl dichlorophosphate, ketoximes, ambient temperature

## Abstract

Upon treatment with phenyl dichlorophosphate (PhOP=OCl_2_) in acetonitrile at ambient temperature, a variety of ketoximes underwent a Beckmann rearrangement in an effective manner to afford the corresponding amides in moderate to high yields.

## 1. Introduction

The Beckmann rearrangement is a well-documented reaction for converting ketoximes into *N*-substituted amides, and serves constantly as a topic of great interest in organic synthesis [1,2,3,4]. However, many conventional Beckmann rearrangement protocols involve drastic conditions and are usually accompanied by environmentally harmful byproducts [5,6]. Thus, enormous efforts have been devoted to the development of more effective and milder versions of this reaction. Along this line, a wealth of useful reagents, including cyanuric chloride (TCT), TAPC, PTSA/ZnCl_2_, BOP-Cl, H_2_NSO_3_H, (EtO)_2_P=OCl, TISC, HgCl_2_, Ru(PPh_3_)_3_(CO)H_2_/dppe/TsOH, BDMS/ZnCl_2_, TsCl, PMSCl, I_2_, and cyclopropenium ion, have been developed to effect the Beckmann rearrangement [7,8,9,10,11,12,13,14,15,16,17,18,19,20,21]. Though these protocols have proved effective, elevated temperatures, however, are usually required in order to obtain satisfactory yields. Recently, supercritical water (scH_2_O) [22,23] was reported to be able to replace both common organic solvents and conventional acid catalysts to facilitate the rearrangement; however, this approach was found synthetically useless in that high temperature and pressure are essential for the reaction to occur. Herein, we wish to report that a convenient new procedure, making use of phenyl dichlorophosphate (**3**) as a key activating agent, has been developed in our laboratories to facilitate the title rearrangement at room temperature.

## 2. Results and Discussion

Phosphorus chemicals are known as versatile activating/dehydrating agents in organic synthesis as demonstrated by many historical cases [24,25,26,27,28,29,30,31,32,33,34]. Though silicaphosphine, prepared from silica gel and trichlorophosphine [35], and trimethylsilyl polyphosphate, prepared from phosphorus pentoxide and hexamethyldisiloxane [36], have been reported as effective promoters for the Beckmann rearrangement, both need to be synthesized prior to the reaction; however, exploration of convention commercially available phosphine-containing reagents, including phenyl dichlorophosphate (**3**), ethyl dichlorophosphate (**4**), diphenyl chlorophosphate (**5**), diethyl chlorophosphate (**6**) [13], and *N*,*N*-dimethylphosphoramidic dichloride (**7**), to induce the same rearrangement at ambient temperature appears to be unknown. Compiled in Table 1 are results of experiments wherein acetophenone oxime (**1**) was used as a model substrate for the screening of various reaction conditions, containing reagents **3**–**7**, respectively, to afford the desired product *N*-phenylacetamide (**2**). Accordingly, when oxime **1** was treated with phosphate **3** in low-polarity solvents, such as CH_2_Cl_2_, toluene, or CH_3_CN, at room temperature, product **2** was obtained in high yields (Entries 1, 3, and 4); among them, CH_3_CN appeared to be the solvent of choice in terms of reaction rate and isolated yield (Entry 4; 1 h, 86%). As well, it was observed that the high-polarity solvents (*i.e.*, THF) were extremely incompatible with **3** to promote the desired 1,2-migration (Entry 2; 3 h, 40%). These significant solvent effects appear ascribable to the hydrochloric acid formed *in situ* during the reaction, a potential migration-inducer whose oxophilicity is much stronger in low-polar solvents, thus making the phenyl chlorophosphate moiety a better leaving group. The intermediacy of hydrochloric acid was further evidenced by the fact that above 1,2-migration process was completely stopped when bases, such as DBU or triethylamine, were added to the initial reaction mixtures with the same equivalents of reagent **3**. Reagent **4** was almost as effective as **3** for the attempted Beckmann rearrangement under similar reaction conditions (Entries 5–7), but in general, an extended reaction time was required as compared to reagent **3**. Other structurally related phosphorus reagents **5**, **6** and **7** in low-polarity solvents were also investigated. In all cases examined (Entries 8–16), they were found to be inferior to phosphate **3** in effecting the 1,2-migration, presumably due to their less active phosphorous centers, potentially caused by a higher delocalization of the electron density and/or more steric hindrance from ligands, as compared to reagent **3**. Moreover, inspired with the fact that Lewis acids could promote Beckmann rearrangement in a catalytic fashion as reported in the literature [7], attempts to combine Lewis acid with reagent **3** to accelerate the reaction rate were then made. Unfortunately, as shown in Table 1 (Entries 17–22), no significant synergistic effects on reaction yield or rate were observed with the addition of Lewis acids as catalyst. However, it was noted that when reagent **5** was combined with either ZnCl_2_ or ZnI_2_ as catalyst (Entries 23 and 24), reaction rates are significantly enhanced along with a slight improvement in yields compared to the corresponding reaction without any catalyst (Entry 8). The underlying cause of these phenomena is still not fully understood and is worthy of further studies. Currently, the reaction system (PhOP=OCl_2_/CH_3_CN/rt) exhibited in Entry 4 was tentatively considered optimum and adopted as a typical procedure. In fact, the gram-scale synthesis of **2** has also been explored and was found to be equally simple and efficient. The above synthetic procedures are detailed in the Experimental section.

To examine the generality of the newly developed protocol, structurally diverse ketoximes were then selected. As listed in Table 2, substrates bearing electron-donating groups in the *para*-position of the phenyl ring (Entries 1–3; 0.5–1 h) were found to undergo 1,2-migration much faster than those with electron-withdrawing functionalities (Entries 6–8; ~13 h), suggesting that when an electron-donating group is present, the ring becomes more electrostatically negative, resulting in the enhancement of the migration rate. Ketoximes, constrained with a six-membered ring such as Entries 5 and 10, required a prolonged reaction time to complete the conversion with relatively lower yields (33−67%), but it appeared to not be problematic in the case of a large-ring system (Entry 11), suggesting that the 1,2-migration process might be susceptible to the ring strain. More interestingly, when the migratory groups are heterocyclic rings, a significant difference in reactivity was observed. A thiophene-containing ketoxime (Entry 9) afforded the desired product within 1.5 h in a high yield of 84%; however, when the corresponding pyridine-containing substrate (Entry 14) was employed, the starting material was recovered intact even when the reaction time was prolonged under refluxing conditions for an additional 6 h. The latter could apparently be attributed to the formation of a hydrochloride salt via protonation of the pyridine nitrogen, rendering the adjacent C-2 migration center extremely positive and thus the migration process was retarded. The aforementioned results also indicate that the migratory aptitude of the alkyl linker appears to be poorer than that of the phenyl ring bearing either an electron-donating or electron-withdrawing substituent (Entries 1–4 and Entries 6–8). Moreover, when different alkyl linkers are present in the ketoxime, the longer ones seem to have a greater migratory aptitude (Entry 12). Several aldoximes were also subjected to above standard conditions, and the corresponding nitriles were isolated in satisfactory yields (70–86%). As well, it is noteworthy that the present methodology can be carried out at ambient temperature with a commercially available reagent, and thus its synthetic utility would be broader than many homogeneous and heterogeneous catalyst systems, particularly the latter in which high temperatures are usually required [37,38,39,40].

**Scheme 1:**
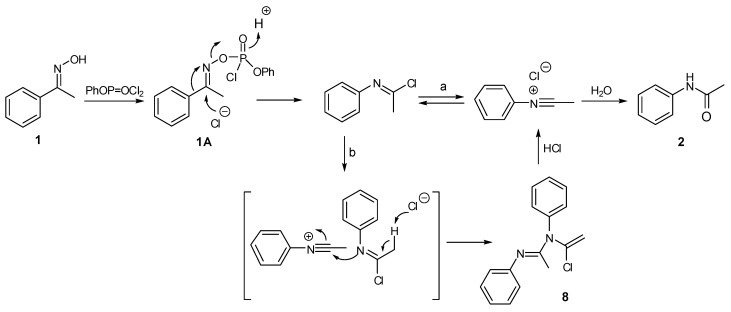
Proposed mechanism of the phenyl dichlorophosphate-induced Beckmann rearrangement of ketoximes.

The proposed mechanism of phenyl dichlorophosphate-induced Beckmann rearrangement is depicted in Scheme 1 using acetophenone oxime (**1**) as a typical example. It is widely accepted that product **2** should be formed following the conventional pathway **a** via intermediate **1A**; however, a shunt pathway **b** cannot be ruled out in that as the reaction was monitored by TLC, product **2** was seen to grow at the expense of a potential intermediate **8** during the progress of reaction, the structure of which was unambiguously identified by an X-ray analysis. A similar TLC behavior, however, was not observed when the same reaction was carried out under refluxing conditions. An explanation for this could be that an extremely rapid conversion of **8** into product **2** (*ca*. 5 min, 91% yield) might take place at elevated temperatures and thus the intermediate was no longer detected by TLC. Dimer **8** thus obtained could also demonstrate the existence of chloroimine and its delocalizing nitrilium ion as proposed in the long-standing accepted pathway **a**.

## 3. Experimental

### 3.1. General

Unless otherwise stated, all materials used were commercially available and used as supplied. Reactions requiring anhydrous conditions were performed in flame-dried glassware, and cooled under an argon or nitrogen atmosphere. Unless otherwise stated, reactions were carried out under argon or nitrogen and monitored by analytical thin layer chromatography performed on glass-backed plates (5 × 10 cm) precoated with silica gel 60 F_254_ as supplied by Merck. Visualization of the resulting chromatograms was done by looking under an ultraviolet lamp (λ = 254 nm) followed by dipping in an ethanol solution of vanillin (5% w/v) containing sulfuric acid (3% v/v) or phosphomolybdic acid (2.5% w/v), and charring by heat gun. Flash chromatography using silica gel 60 of 230−400 mesh size as supplied by Merck was used routinely for purification and separation of product mixtures. Eluent systems are given in volume/volume concentrations. ^1^H-NMR and ^13^C-NMR spectra were recorded at 400/600 and 100/150 MHz, respectively, on a Bruker Avance 400 FT-NMR or Bruker DMX-600 instrument. Chloroform-*d* or dimethyl sulfoxide-*d*_6_ was used as the solvent and TMS (δ 0.00 ppm) as an internal standard. Chemical shift values are reported in ppm relative to the TMS in delta (δ) units. Coupling constants (*J*) are expressed in Hz. High resolution electron impact mass spectra (HRMS (EI)) were recorded using a FINNIGAN MAT-95XL mass spectrometer. Spectral data were recorded as *m/z* values. Combustion elemental analyses using Heraruc CHN-O Rapid Elemental Analyzer were performed by the microanalytical laboratory at National Chung Hsing University, Taiwan. Crystallographic data can be obtained free of charge via www.ccdc.cam.ac.uk/conts/retrieving.html or deposite@ccdc.cam.ac.uk using CCDC No.: 690116.

### 3.2. General Procedure for the Synthesis of Compounds in Table 2

Phenyl dichlorophosphate (**3**, 0.633 g, 3 mmol) was added dropwise at room temperature to a solution of acetophenone oxime (**1**, 0.270 g, 2 mmol) in anhydrous acetonitrile (4 mL). The resulting mixture was stirred at the same temperature for *ca*. 1 h, and then saturated sodium bicarbonate solution (5 mL) was added to quench the reaction. The aqueous layer was separated and extracted with ethyl acetate (3 × 10 mL). The combined organic extracts were washed with brine, dried over MgSO_4_, filtered and concentrated to give the crude product, which was purified by flash chromatography on silical gel (30% EtOAc in *n*-hexane) to afford amide **2** (0.232 g, 86%) as a white solid.

*N-Phenylacetamide* (**2**). A white solid; m.p. 114–115 °C; ^1^H-NMR (CDCl_3_) δ 8.27 (brs, 1H, NH), 7.50 (d, *J* = 7.9 Hz, 2H), 7.27 (d, *J* = 7.6 Hz, 2H), 7.07 (t, *J* = 7.4 Hz, 1H), 2.12(s, 3H); ^13^C-NMR (CDCl_3_) δ 169.2, 138.2, 129.0, 124.4, 120.3, 24.5; Anal. Calcd. for C_8_H_9_NO: C 71.29, H 6.71, N 10.36; Found: C 71.09, H 6.71, N 10.26.

*N-(4-Methylphenyl)acetamide* (Table 2, Entry 1). A white solid; m.p. 149–150 °C; ^1^H-NMR (CDCl_3_) δ 7.74 (brs, 1H, NH), 7.37 (d, *J* = 7.8 Hz, 2H), 7.08 (d, *J* = 7.8 Hz, 2H), 2.29 (s, 3H), 2.12 (s, 3H); ^13^C-NMR (CDCl_3_) δ 168.8, 135.6, 134.0, 129.6, 120.4, 24.6, 21.0. Anal. Calcd. for C_9_H_11_NO: C 72.46, H 7.43, N 9.39; Found: C 72.73, H 7.47, N, 9.08.

*N-(4-Methoxyphenyl)acetamide* (Table 2, Entry 2). A brown solid; m.p. 127–129 °C; ^1^H-NMR (CDCl_3_) δ 7.67 (brs, 1H, NH), 7.38 (d, *J* = 8.8 Hz, 2H), 6.82 (d, *J* = 8.8 Hz, 2H), 3.77 (s, 3H), 2.12 (s, 3H); ^13^C-NMR (CDCl_3_) δ 168.7, 156.6, 131.3, 122.2, 114.3, 55.6, 24.4; Anal. Calcd. for C_9_H_11_NO_2_: C 65.44, H 6.71, N 8.48; Found: C 65.31, H 6.86, N 8.13.

*N-(2,3-Dihydro-1,4-benzodioxin-6-yl)acetamide* (Table 2, Entry 3). A colorless oil; ^1^H-NMR (CDCl_3_) δ 7.08 (brs, 1H, NH), 7.09 (s, 1H), 6.85–6.76 (m, 2H), 4.20 (m, 4H), 2.11 (s, 3H); ^13^C-NMR (CDCl_3_) δ 168.2, 143.4, 140.5, 131.5, 117.1, 113.7, 109.9, 64.4, 64.2, 24.4; HRMS (FAB) calcd for C_10_H_1__2_NO_3_ (M+H)^+^: 194.0817; Found: 194.0829.

*N-(2-Naphthyl)acetamide* (Table 2, Entry 4). A colorless oil; ^1^H-NMR (400 MHz, CDCl_3_) δ 8.16 (s, 1H), 7.94 (brs, 1H, NH), 7.75–7.70 (m, 3H), 7.46–7.24 (m, 3H), 2.19 (s, 3H); ^13^C-NMR (100 MHz, CDCl_3_) δ 169.1, 135.6, 134.0, 130.8, 128.9, 127.7, 126.6, 125.1, 120.2, 117.0, 24.8; Anal. Calcd. for C_12_H_11_NO: C 77.81, H 5.99, N 7.56; Found: C 77.80, H 5.89, N 7.68.

*1,2,3,4-Tetrahydro-7-methoxy-5H-benzazepin-2-one* (Table 2, Entry 5). A colorless oil**;** ^1^H-NMR (CDCl_3_) δ 7.63 (m, 1H), 7.12 (brs, 1H, NH), 6.80 (m, 1H), 6.68 (s, 3H), 3.80 (s, 3H), 3.11–1.95 (m, 6H); ^13^C-NMR (CDCl_3_) δ 174.1, 161.6, 140.6, 130.7, 127.4, 114.2, 111.8, 55.2, 39.7, 30.7, 30.4; HRMS (FAB) calcd for C_11_H_1__4_NO_2_ (M+H)^+^: 192.1025; Found: 192.1021.

*N-(4-Chlorophenyl)acetamide* (Table 2, Entry 6). A colorless oil; ^1^H-NMR (DMSO-*d_6_*) δ 10.1 (brs, 1H, NH), 7.60 (d, *J* = 7.8 Hz, 2H), 7.32 (d, *J* = 7.8 Hz, 2H), 2.04 (s, 3H); ^13^C-NMR (DMSO-*d_6_*) δ 168.4, 138.3, 128.5, 126.5, 120.5, 24.0; Anal. Calcd. for C_8_H_8_ClNO: C 56.65, H 4.75, N 8.26; Found: C 56.70, H 4.92, N 8.12.

*N-(4-Nitrophenyl)acetamide* (Table 2, Entry 7). A colorless oil; ^1^H-NMR (DMSO-*d_6_*) δ 10.5 (brs, 1H, NH), 8.20 (d, *J* = 9.2 Hz, 2H), 7.81 (d, *J* = 9.2 Hz, 2H), 2.11 (s, 3H); ^13^C-NMR (DMSO-*d_6_*) δ 169.3, 145.4, 142.0, 125.0, 118.5, 24.2; Anal. Calcd for C8H8N2O2: C 53.33, H 4.48, N 15.55; Found: C 53.08, H 4.55, N 15.24.

*N-(2-Bromophenyl)acetamide* (Table 2, Entry 8). A yellow solid: m.p. 98–99 °C; ^1^H-NMR (DMSO-*d_6_*) δ 9.45 (brs, 1H, NH), 7.65–7.10 (m, 4H), 2.07 (s, 3H); ^13^C-NMR (DMSO-*d_6_*) δ 168.5, 136.5, 132.6, 127.9, 127.2, 126.8, 117.8, 23.3; Anal. Calcd for C_8_H_8_BrNO: C 44.89, H 3.77, N 6.54; Found: C 44.96, H 3.75, N 6.33.

*N-(2-Thienyl)acetamide* (Table 2, Entry 9). A colorless oil; ^1^H-NMR (DMSO-*d_6_*) δ 11.1 (brs, 1H, NH), 6.90–6.61 (m, 3H), 2.05 (s, 3H); ^13^C-NMR (DMSO-*d_6_*) δ 166.2, 139.9, 123.5, 116.6, 110.3, 22.5; Anal. Calcd for C_6_H_7_NOS: C, 51.04; H, 5.00; N, 9.92; S, 22.71; Found: C, 50.92; H, 5.29; N, 9.66; S, 22.50.

*ε-Caprolactam* (Table 2, Entry 10). A colorless oil; ^1^H-NMR (CDCl_3_) δ 7.11 (brs, 1H, NH), 3.14–3.11 (m, 2H), 2.39–2.36 (m, 2H), 1.68–1.57 (m, 6H); ^13^C-NMR (CDCl_3_) δ 179.5, 42.8, 36.8, 30.7, 29.8, 23.3; Anal. Calcd for C_6_H_11_NO: C 63.68, H 9.80, N 12.38; Found: C 63.60, H 9.72, N 12.43.

*Azacyclotridecan-2-one* (Table 2, Entry 11). A colorless oil; ^1^H-NMR (DMSO-*d_6_*) δ 7.86 (brs, 1H, NH), 3.07–3.03 (m, 2H), 2.06–2.03 (m, 2H), 1.55–1.24 (m, 18H); ^13^C-NMR (DMSO-*d_6_*) δ 172.7, 37.8, 35.4, 27.7, 26.3, 25.8, 25.7, 25.5, 24.8, 24.5, 24.2, 23.5; HRMS (FAB) calcd for C_12_H_2__4_NO (M+H)^+^: 198.1858; Found: 198.1858.

*N-Heptylacetamide* (Table 2, Entry 12). A colorless oil; ^1^H-NMR (CDCl_3_) δ 6.20 (brs, 1H, NH), 3.20 (s, 3H), 1.35–1.21 (m, 12H), 0.91–0.79 (m, 3H); ^13^C-NMR (CDCl_3_) δ 170.7, 39.9, 31.7, 29.7, 29.4, 28.9, 26.8, 22.5, 14.0; HRMS (EI) calcd for C_9_H_20_NO (M+H)^+^: 158.1545; Found: 158.1547.

*N-Pentylhexanamide* (Table 2, Entry 13). A colorless oil; ^1^H-NMR (DMSO-*d_6_*) δ 7.76 (brs, 1H, NH), 3.03–2.98 (m, 2H), 2.04–2.01 (m, 2H), 1.50–1.20 (m, 12H), 0.86–0.82 (m, 6H); ^13^C-NMR (DMSO-*d_6_*) δ 170.0, 38.4, 35.4, 31.0, 28.9, 28.7, 25.1, 22.1, 22.0, 13.7; HRMS (FAB) calcd for C_11_H_2__4_NO (M+H)^+^: 186.1858; Found: 186.1857.

*N-(1-Chlorovinyl)-N,N'-diphenylacetamidine* (**8**). A white solid; m.p. 90–92 °C; ^1^H-NMR (CDCl_3_) δ 7.44–7.39 (m, 4H), 7.30–7.26 (m, 3H), 7.04–7.00 (m, 1H), 6.81–6.79 (m, 2H), 5.45 (d, *J* = 1.2 Hz, 1H), 5.38 (d, *J* = 1.2 Hz, 1H), 1.93 (s, 3H); ^13^C-NMR (CDCl_3_) δ 155.4, 150.3, 142.3, 140.0, 129.3, 128.8, 126.9, 126.8, 122.6, 130.0, 114.6, 16.5; HRMS (FAB): calcd for C_16_H_1__6_N_2_Cl (M+H)^+^: 271.1002; Found: 271.1003.

### 3.3. Gram-Scale Synthesis of Amide ***2***

A mixture of acetophenone oxime (**1**, 13.5 g, 100 mmol) and phenyl dichlorophosphate (**3**, 31.6 g, 150 mmol) was stirred in CH_3_CN (200 mL) for 1 h, product **2** was obtained in 88% yield (11.9 g) as a white solid after the usual work-up procedure and further recrystallization (ethyl acetate/*n*-hexane = 5:1).

## 4. Conclusions

In summary, we have developed a mild and effective reaction system to induce the Beckmann rearrangement of ketoximes at ambient temperature, leading to the corresponding amides in moderate to high yields. It is believed that the title system will be a valuable addition to the synthetic organic chemistry methodology in terms of its operational simplicity scale-up practicability and use of readily accessible phosphorous reagents.

## Figures and Tables

**Table 1:**
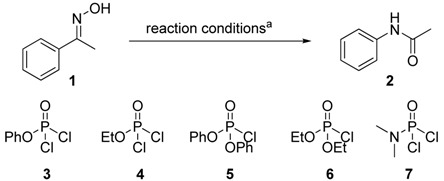
Beckmann rearrangement of acetophenone oxime promoted by phosphorous reagents under various reaction conditions.

Entry	Reagent	Catalyst ^b^	Solvent	Time (h)	Yield (%) ^c^
1	3	−	CH_3_CN	3	83
2	3	−	THF	3	40
3	3	−	Toluene	16	87
4	3	−	CH_3_CN	1	86
5	4	−	CH_2_Cl_2_	6	76
6	4	−	Toluene	14	74
7	4	−	CH_3_CN	2	81
8	5	−	CH_2_Cl_2_	20	72
9	5	−	Toluene	20	70
10	5	−	CH_3_CN	5	80
11	6	−	CH_2_Cl_2_	24	60
12	6	−	Toluene	26	54
13	6	−	CH_3_CN	7	61
14	7	−	CH_2_Cl_2_	30	55
15	7	−	Toluene	30	50
16	7	−	CH_3_CN	8	52
17	3	ZnCl_2_	CH_2_Cl_2_	1	85
18	3	ZnCl_2_	CH_3_CN	1	81
19	3	ZnI_2_	CH_2_Cl_2_	0.6	84
20	3	ZnI_2_	CH_3_CN	0.8	79
21	3	BCl_3_	CH_2_Cl_2_	0.8	82
22	3	BCl_3_	CH_3_CN	0.8	80
23	5	ZnCl_2_	CH_2_Cl_2_	5	79
24	5	ZnI_2_	CH_2_Cl_2_	3	83

^a^ All reactions were performed using oxime **1** (2 mmol) and phosphorous reagent (3 mmol) in 4 mL of solvent as indicated above at room temperature; ^b^ 10 mol% of catalyst was added; ^c^ Yields are for isolated, chromatographically pure products.

**Table 2:**
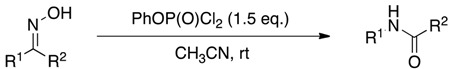
Conversion of ketoximes into amides under treatment with phenyl dichlorophosphate.

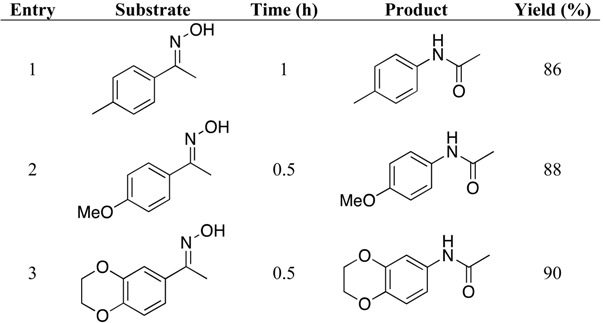 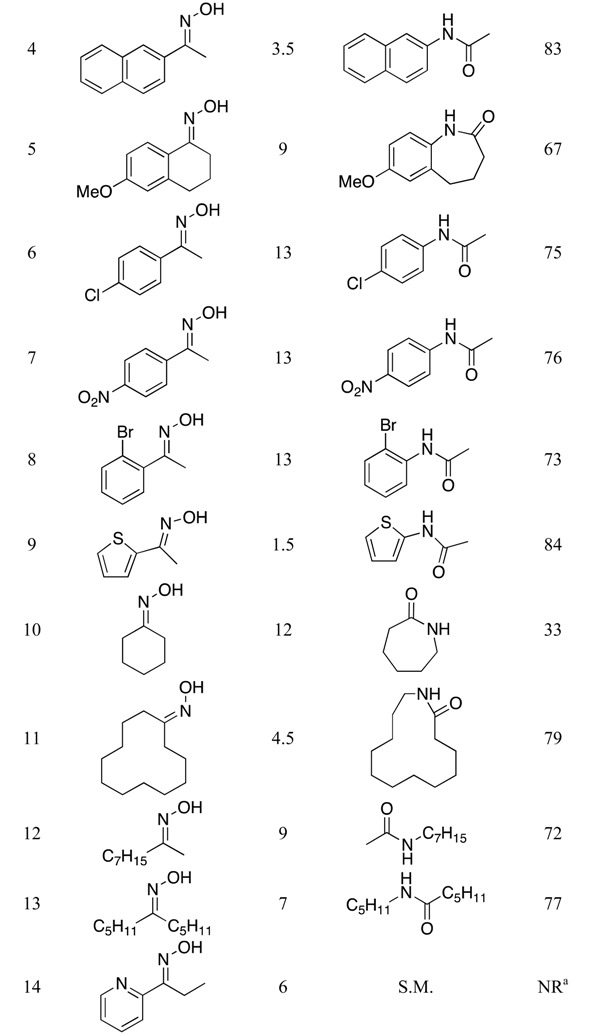

^a^ No reaction occurred at room temperature or under refluxing conditions.
